# Management strategies and clinical follow-up of pregnant women with intracranial meningioma

**DOI:** 10.25122/jml-2021-0012

**Published:** 2021

**Authors:** Andreea Elena Dumitru, Anca Panaitescu, George Iancu, Francesca Gabriela Paslaru, Alexandru Catalin Paslaru, Radu Mircea Gorgan, Gheorghe Peltecu

**Affiliations:** 1. Obstetrics and Gynecology Department, Clinical Hospital of Obstetrics and Gynecology “Filantropia”, Bucharest, Romania; 2. Department of Obstetrics and Gynecology, “Carol Davila” University of Medicine and Pharmacy, Bucharest, Romania; 3. Neurosurgical Department, “Bagdasar-Arseni” Clinical Emergency Hospital, Bucharest, Romania; 4. Department of Functional Sciences, “Carol Davila” University of Medicine and Pharmacy, Bucharest, Romania

**Keywords:** meningioma, pregnancy, progesterone receptor, visual impairment

## Abstract

Meningiomas are common primary tumors of the central nervous system. The incidence at the age of fertility is low, although there are some hormonal mechanisms involved. Growth in size was observed during the luteal phase of the menstrual cycle, which could lead to developing new symptoms during pregnancy or worsening of the already existing ones. Visual impairment is the chief complaint, followed by headache, nausea, vomiting, and seizures. Diagnosis is based on neurological examination, ophthalmoscopy, imaging techniques like gadolinium-enhanced magnetic resonance imaging (MRI), and contrast-enhanced computed tomography (CT) scans, bearing in mind the patient’s irradiation and prejudice on the fetus together with the histopathological examination. The objective of the review is to determine the influence of meningioma on pregnancy and vice-versa and provide a strategy of follow-up for maternal-fetal specialists and not only. We performed a systematic review by searching relevant information in PubMed and Wiley databases using keywords as meningioma, pregnancy, progesterone receptors. The results showed that besides a similar incidence of meningioma in pregnant and non-pregnant women, symptoms might flare during pregnancy due to water retention, engorgement of vessels, and the presence of sex hormone receptors on tumor cells. Meningioma may impact the route of pregnancy with adverse effects on the fetus. Thus, fetal monitoring by biophysical profile and cardiotocography (CTG) is needed. The preferred treatment option is surgery, but gestational age and the woman’s status must be taken into consideration.

## Introduction

Meningiomas are common primary tumors of the central nervous system, predominantly affecting women between the ages of 40–60 and postmenopausal women [1]. There is a low incidence at the age of fertility, although there are some hormonal mechanisms involved in their growth and development [2], an aspect which was provided by epidemiological studies showing a female to male ratio of approximately two or three to one [3–6]. Growth in size was observed during the luteal phase of the menstrual cycle, which could lead to developing new symptoms during pregnancy or worsening of the already existing ones. [7]. Some tumor features were found to be of high statistical significance during pregnancy, as compared to their prevalence in the general population, including the location in the parasellar region, the blood supply from the anterior circulation, presence of visual impairment, choroid morphology, and high rate of clear-cell, suggesting that dramatic flare-ups in size sometimes observed during pregnancy could be related to hormonal influence from the pituitary gland, especially elevated prolactin levels [8].

Large, aggressive, highly symptomatic meningiomas require emergency surgery, regardless of the pregnancy status. However, this is a rare case scenario. Most of the time, the growth in size is slow, with no severe impact on maternal or fetal life and normal pregnancy route under monitoring. The timing of the neurosurgical intervention is still a matter of debate.

## Material and Methods

We performed a systematic review by searching databases of PubMed and Wiley for relevant information using keywords such as meningioma, pregnancy, progesterone receptors. From the articles reviewed, we excluded the ones on spinal meningioma or those that presented irrelevant information regarding pregnancy and their influence on it.

## Results

### Intracranial meningioma effects on pregnancy

The incidence of meningioma in pregnant women is comparable with that in non–pregnant women of the same age group. However, symptoms may burst during pregnancy. This has been attributed to water retention, engorgement of vessels, and the presence of sex hormone receptors on tumor cells, leading to the explosive growth of the tumor [8].

When suspected during pregnancy, these tumors can be assessed by a neurological examination. If the patient is stable, with no severe neurological deficit, a close follow-up along with a magnetic resonance imaging (MRI) test would be sufficient so that the pregnancy may continue through its usual course. The maternal health status and pregnancy prognosis should be the primary area of concern when evaluating such patients. The mode of delivery should be discussed previously in a team of specialists, including an obstetrician, an anesthesiologist, and a neurologist. C-section is preferred and considered safer in preventing the stress induced by the Valsalva maneuver, which may further increase intracranial pressure (ICP) [1].

### The effect of pregnancy on meningioma

These tumors tend to grow slowly; however, pregnancy seems to speed up this process creating symptoms, by involving hormone-related mechanisms. The role of the progesterone receptor (PR) has been studied in numerous papers. There is an inverse relationship between protein expression and tumor grade [11]. While mitosis is increasing with higher grade, meningiomas in pregnancy are predominantly low grade (WHO I) with negligible mitotic activity. Still, the role of progesterone is far from being fully understood [12, 13]. Considering the fact that this tumor growth is usually more rapidly occurring in the luteal phase of the menstrual cycle or in the second or third trimester of pregnancy when progesterone plasma concentration is higher, this could be due to the effect of sex hormones [14]. Contradictory arguments consist of progesterone receptors being expressed by tumors found in male patients and the pediatric population as well. Moreover, their peak of incidence is far beyond the fertile age when the serum level of progesterone is relatively low. Furthermore, long-term hormone replacement therapy also raises the incidence of disease, although such correlation has not been shown after the use of oral contraceptives [15].

Prolactin receptors have also been found in meningiomas and are closely associated with the growth rate [1]. Due to the abundance of prolactin receptors that produce physiological pituitary hyperplasia, meningiomas are more likely to grow during and after pregnancy. Also, these tumors may cause compression of the optic nerve and chiasm, resulting in visual impairment [20].

### Fetal monitoring

Most cases undergo noninvasive fetal monitoring using ultrasound to monitor the amniotic fluid volume (AFV) - a normal value considered with one pocket larger than 3 cm, the fetal breathing movements (FBM) - more than 1 FBM/ 30minutes, the fetal movements (FM), namely body/limb movements - more than 4 FM/ 30 minutes, the fetal tone (FT) - measuring active extension, flexion, opening and closing of the hands together with a cardiotocography (CTG) showing more than 2 accelerations and palpated fetal movements/ 20 minutes [12].

### Timing of surgical intervention

A meningioma diagnosed during pregnancy causes the surgeon to ponder whether to perform surgery immediately or wait for the end of the pregnancy. If the patient presents with a severe neurological disfunction or the size of the tumor could produce intracranial herniation, it is considered an emergency, and surgery must be performed. The difficulty arises in cases of advanced gestational age, when fetal prejudice may result.

A surgical excision without the termination of pregnancy must be handled carefully, taking into consideration intraoperative blood loss, hypotension, hypovolemia, and hypoxia. Corticosteroids may be helpful in treating severe edema perioperatively or peripartum. The preferred choice is a divided dose of 2–4 mg dexamethasone every 6 hours. Since mannitol is able to pass through the maternal-fetal barrier, it should only be used under urgent conditions. Monotherapy should be the elected therapy for seizures. Women with a history of seizures should take folic acid vitamin-K1 at the early stages of pregnancy to prevent the risk of neural tube defects [9, 10].

In gestational age younger than 10–12 weeks, when organogenesis is still incomplete, anesthetic agents along with anti-epileptic and anti-edema treatments may cause adverse effects on the fetus [1]. The multidisciplinary team should offer proper advice and information to the family, together with genetic counseling, and a therapeutic abortion would be advised.

In the case of gestational age greater than 12 weeks, when organogenesis is believed to be complete, neurosurgical intervention along with close fetal monitoring could be proposed if the obstetric examination reveals a healthy fetus. In cases where fetal lung maturation is complete, or a neonatal intensive care unit is present, a 32–33 weeks pregnancy with the consent of the family and an obstetrician may undergo a dual surgery where a C-section along with the excision of the lesion would be performed.

Priddy *et al.* published an algorithm for the management of non-urgent but symptomatic skull base tumors in the obstetric population and stated that the most critical factor in evaluating the need for urgent surgical management is the trimester of pregnancy, with the second trimester being associated with the lowest risk of spontaneous abortion or preterm birth, as compared to the first and third trimester, respectively [21].

In general, babies born at 24–25 weeks have a survival rate of approximately 50%, compared to 50–70% survival rates for babies born at 26–27 weeks and roughly 90% survival rates for babies born at 27 weeks (weighting 900–1,000 g or more) [22].

Laviv *et al.* performed a systematic review on clinical management of intracranial meningiomas diagnosed either during pregnancy or immediately postpartum and compared the outcomes of patients who underwent surgical resection during pregnancy to those who were operated on during the postpartum period, all patients being symptomatic at the time of diagnosis. They found preterm births (defined as delivery before week 37) to occur at similar rates between the two groups, all of them being associated with a C-section, but with significantly more patients subjected to surgery antepartum undergoing emergency C-sections for either severe neurological symptoms (5.71%), sudden eclampsia (2.85%), metabolic acidosis (2.85%), macrosomia (2.85%), persistent fetal bradycardia during the neurosurgical intervention (2.85%), lack of progress after labor induction (2.85%) or bradycardia during labor (2.85%). The risk of maternal death was higher in the prenatal surgical intervention group, with two deaths (5.71%) reported: one patient who deteriorated rapidly during labor because of increased intracranial pressure and one patient who underwent a C-section and craniotomy in the same session but suffered from uncontrolled status epilepticus 3 days later. No maternal deaths were reported in the postnatal surgical intervention group. Regarding fetal outcomes, two deaths (5.71%) were reported: one patient underwent a spontaneous abortion at week 26, and another patient suffered from persistent fetal bradycardia during the neurosurgical intervention, resulting in an emergency C-section at week 26 with perinatal asphyxia and death 14 days later [23].

## Discussion

### How to diagnose?

When establishing the diagnosis, several steps should be followed for accurate interpretation.

**Figure 1.**
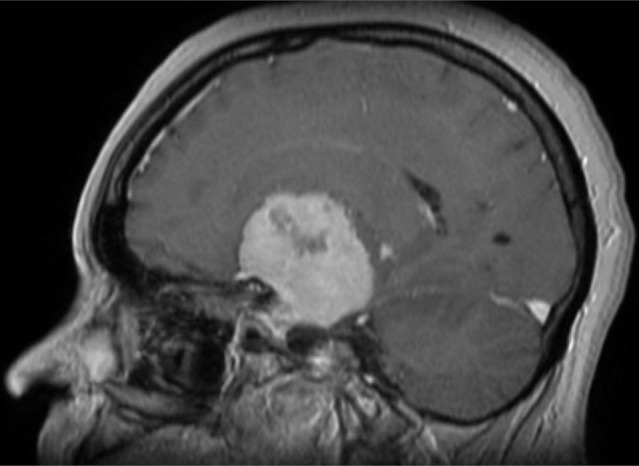
Gadolinium-enhanced T1-weighted MRI scan, sagittal section, showing a large extra-axial tumor inserted on the middle skull base dura mater, with the specific dural tail sign, suggestive of meningioma.

**Figure 2.**
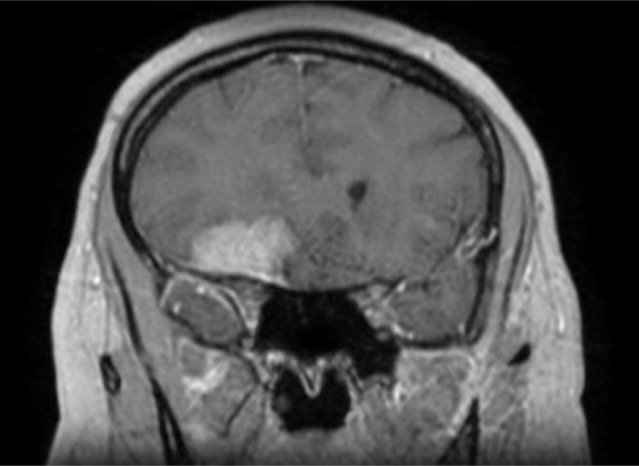
Gadolinium-enhanced T1-weighted MRI scan, coronal section, showing a large extra-axial tumor inserted on the dura mater of the right sphenoid wing, with the specific dural tail sign, suggestive of sphenoid wing meningioma.

Firstly, the detailed neurological examination has an important role in deciding over most frequent symptoms such as headache, dizziness, focal signs, visual acuity impairment, and epileptic seizures, along with ophthalmoscopy. Imaging techniques have an essential role, preferably the use of an MRI scan. In cases of extreme necessity, when irradiation cannot be avoided, a CT scan could be performed for better observation of the bony structures. Due to variations in size and location, meningiomas can have different clinical presentations. In most cases, they present as slow-growing tumors and can often remain asymptomatic for a long time. Sphenoid wing meningioma (Figures 1, 2) can cause unilateral visual deficit and visual acuity loss [16]. When located in the parasellar region, these tumors can produce bilateral visual loss, which can be exacerbated when the pituitary gland enlarges during pregnancy [17].

Current literature shows that visual impairement is the chief complaint, followed by headache, nausea, vomitting and seizures [18]. Meningioma can arise from any intracranial or spinal dural surface. The increase in intracranial pressure (ICP) results in reduced venous return, producing a blurry and oedematous papilla on optic funduscopy, toghether with nausea, vomitting, confusion that may be misdiagnosed as hyperemesis gravidarium, but also severe outcome with the patient falling into coma as a consequence of transtentorial herniation and brainstem compression [18]. Afecting the oculomotor nerve causes functional deficit of extraocular muscles, anisocoria, pupillary dilatation and absence of light reaction.

**Figure 3.**
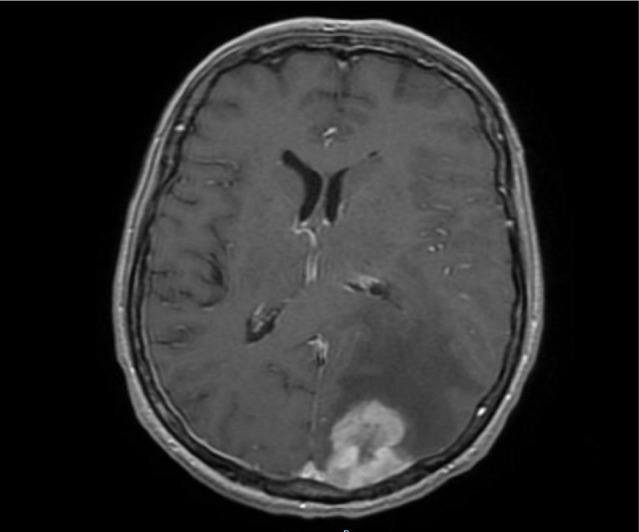
Gadolinium-enhanced T1-weighted MRI scan, axial section, showing a large left parieto-occipital extra-axial tumor, with a particular dural tail sign and peritumoral edema compressing the occipital horn of the left lateral ventricle and causing midline shift, suggestive of meningioma.

**Figure 4.**
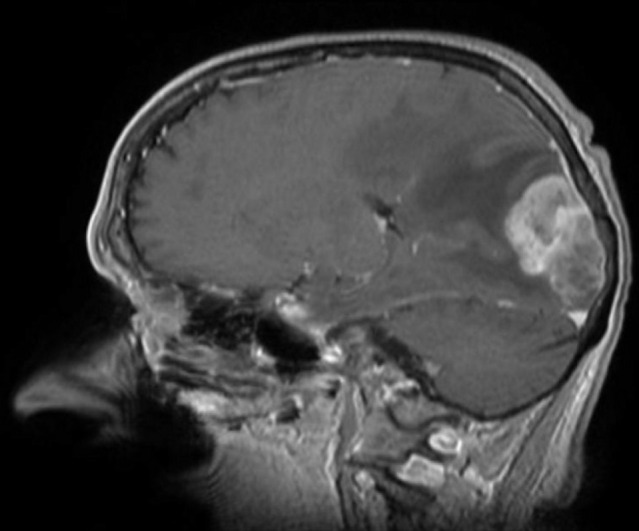
Gadolinium-enhanced T1-weighted MRI scan, sagittal section, showing a large left parieto-occipital extra-axial tumor, with a characteristic dural tail sign and peritumoral edema, suggestive of meningioma.

Secondly, the use of imaging techniques is essential. During pregnancy, MRI is the preferred choice because X-ray has teratogenic effects. Typical aspect is better observed on T1-weighted sequences under the form of an extra-axial lesion with a characteristic dural tail sign and perifocal edema (Figures 3, 4), compressing nearby structures (e.g. corpus callosum and the lateral ventricles). Definitive diagnosis can only be established after histopathological examination of tissue sample obtained by either surgical resection or tumor biopsy, together with imunohistochemistry, where positive progesterone receptors can be found, suggesting a mechanism by which neurological symptoms can flare during pregnancy, in pre-existing asymptomatic meningiomas, due to increased hormone levels. Moreover, it was observed that there is an increased incidence of these tumors in patients who undergo diagnostic or therapeutic irradiation, so the use of some imaging techniques should be carefully evaluated [19].

### Treatment

Although stereotactic radiosurgery can be a treatment option, surgery is usually the preferred choice for the treatment of meningiomas because it provides a rapid reduction in size and a tumor sample for histopathological examination. Pharmaceutical therapy aims to reduce brain edema and halt its progression. Management strategy for brain tumors during pregnancy should be judged to the individual case. Fetal biophysical profile scoring system, Umbilical arterial Doppler assessment, neuroanesthesia, and microsurgical techniques permit safe neurosurgical management of brain tumors during pregnancy. Emergency surgical intervention should be reserved for patients with fast tumor growth rates, active hydrocephalus requiring shunting, signs of impending herniation, or progressive neurological deficits.

## Conclusion

Meningiomas are slow-growing tumors arising from arachnoid cap cells, determining symptoms dependent on the location and pressure exerted on the nearby tissues. Most cases are sporadic, but risk factors include neurofibromatosis type 2 and environmental factors such as previous head and neck irradiation.

Occasionally, they may present with an acute onset showing a suddenly decreased level of consciousness, seizures, or unexpected visual loss, which can state for a differential diagnosis with eclampsia, meningitis, intracranial hemorrhage or drug ingestion, more frequent than meningiomas.

Advances in noninvasive fetal monitoring using ultrasound, combined with a close and regular clinical and neurological evaluation of the mother, allow most of these pregnancies to continue safely to term and finish with safe delivery.

Meningioma may impact the route of pregnancy with adverse effects on the fetus; thus, fetal monitoring by biophysical profile and CTG is needed. The preferred treatment option is surgery, but gestational age and the woman’s status must be taken into consideration before.

## Acknowledgments

### Conflict of interest

The authors declare that there is no conflict of interest.
